# Massive thymic hyperplasia in a 15‐month‐old boy: Case report and literature review

**DOI:** 10.1002/ccr3.1896

**Published:** 2018-11-10

**Authors:** Elisa Tadiotto, Maria Clemente, Luca Pecoraro, Giorgio Piacentini, Daniela Degani, Angelo Pietrobelli

**Affiliations:** ^1^ Paediatric Clinic, Department of Life and Reproduction Sciences Azienda Ospedaliera Universitaria Integrata Verona Italy; ^2^ Pennington Biomedical Research Center Baton Rouge Louisiana

**Keywords:** computerized tomography scan, pediatric, thymectomy, true thymic hyperplasia

## Abstract

A surgical approach is the choice in young infants with MTH, who are furthest from the time of physiological involution of the thymus, and when the thymus achieves the largest relative size, a surgical approach is the choice. Steroid therapy has been shown to be ineffective (4, 9, 16, 18‐20). No surgical complications have been reported, and the outcome is excellent. Recurrence has been seen in only one case.

## 1 CASE REPORT

**Figure 1:**
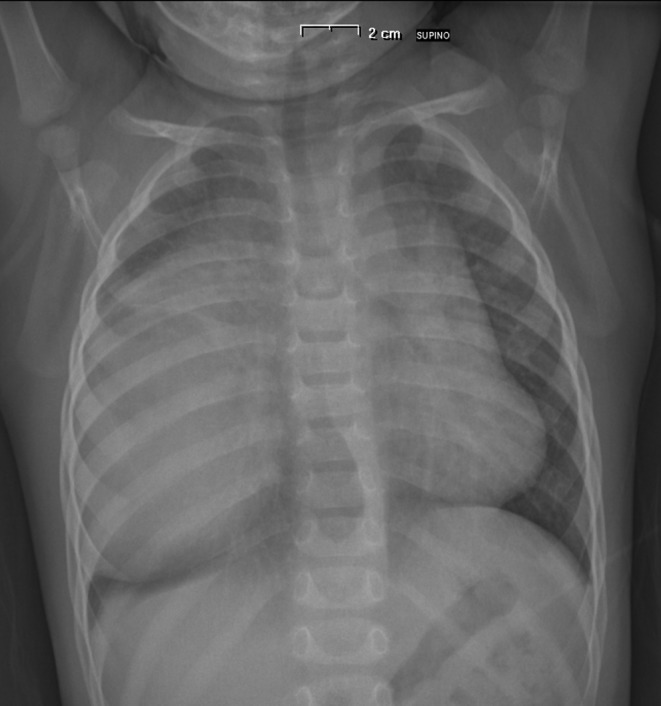
Chest X‐ray of the infant at presentation, showing a large intrathoracic radiopaque mass occupying the right hemithorax

**Figure 2:**
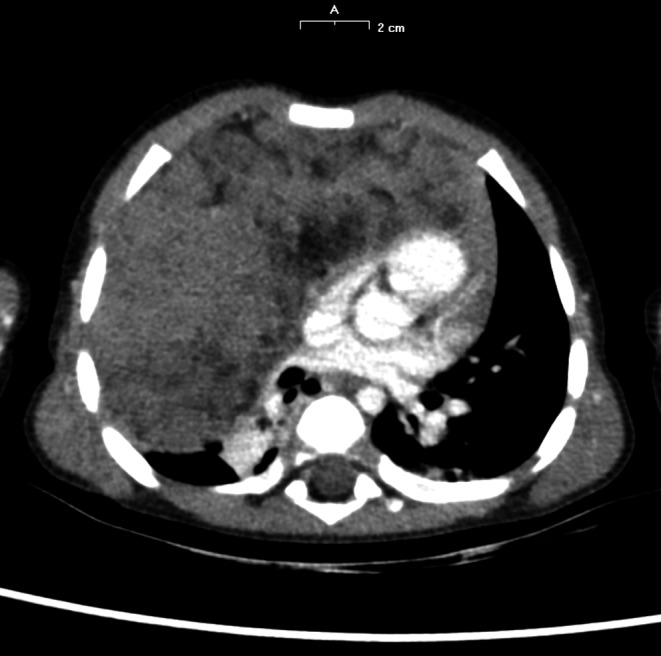
Chest CT scan showing the large mediastinal mass

A 15‐month‐old boy was admitted to the Emergency Room with a history of 3 days of fever, cough, and suspected pneumonia. In his past history, only a slight decline in appetite was reported. Physical examination revealed diminished air entry and crackles at the right hemithorax. A chest X‐ray showed a large intrathoracic radiopaque thickening occupying the right hemithorax (Figure 1). Laboratory tests revealed: leukocyte count 16.36 × 10^9^/L (of which 74% lymphocytes), normal C‐reactive protein, and normal biochemical profile. He was admitted to our pediatric clinic and treated with ceftriaxone (80 mg/kg/d) and clarithromycin (15 mg/kg/d). A chest X‐ray after 5 days of treatment revealed an improvement in the thickening of the right lung, but persistence of mediastinal enlargement. A chest computerized tomography scan (CT scan) was done and showed enlargement of the anterior mediastinum, occupied by solid inhomogeneous predominantly hypodense hypovascularized tissue, with a total dimension of 9.6 × 6 × 10 cm, with left paramedian development with minimal imprint on the jugulo‐subclavian confluence and on the homolateral anonymous vein. This was associated with pleural effusion of a maximum thickness of about 7 mm. The thymus was not well recognized (Figure 2).

Abdominal and neck ultrasounds were normal. Echocardiography showed minimum pericardial effusion but no heart chamber compression by the mediastinal mass. Oncologic markers (α‐fetoprotein, vanillylmandelic acid, human chorionic gonadotropin, urinary vanillylmandelic acid, homovanillic acid and 5‐hydroxic‐indoleacetic acid) were negative. Subsequent complete blood count revealed an increase in lymphocytosis (81.9% of 21.78 × 10^9^/L leukocytes).

A percutaneous biopsy was carried out to exclude malignancy. Fragment analysis was compatible with thymic tissue, making the diagnosis of true thymic hyperplasia (TTH).

Because of clinical improvement and radiological stability of the size of the mass, the patient was discharged. A steroidal therapy (prednisone 2.5 mg/kg/d) was prescribed for 40 days. Radiological investigations showed initially minimal change in tumor size, but subsequently a rapid increase in dimension that reached 10.8 × 11 × 9 cm, with compression of the heart chamber and right main bronchus with atelectasis. The child underwent surgical excision of the mass via right thoracotomy. The mass weighed 492 g and measured 15 × 11.5 × 5 cm. Microscopic finding showed preservation of the normal thymic architecture. Immunohistochemical analysis revealed TTH associated with extramedullary myelopoiesis. Postoperative recovery was uneventful, and the patient was discharged from hospital five days after surgery. Laboratory results (complete blood count and inflammatory indices) at 1 month were normal. A chest X‐ray 3 months after surgery was normal. At 10 months of follow‐up, the child was asymptomatic and showed regular growth; no recurrence was detected.

## 2 DISCUSSION AND LITERATURE REVIEW

The thymus is a gland situated in the anterior mediastinum, embryologically derived from the third and fourth pairs of pharyngeal pouches.^1^, ^2^, ^3^ Its size varies with age. From a birth mean weight of 15 g, the thymus grows in size until puberty to a mean weight of 30‐40 g,^4^, ^5^ and then, it undergoes progressive atrophy, to no more than 5‐15 g in the elderly.^3^ In early infancy, the thymus reaches its largest relative size, because its rate of increase is less than the rest of the body in a growing child.^1^, ^6^ After the age of 2 years, the thymic shadow is less frequently visible.^6^

True thymic hyperplasia is a rare but significant pathology in pediatric age because of its potentially serious consequences. It is characterized by an increase in size and weight of the thymus gland with preservation of normal thymic architecture and immunohistochemical appearance, but without an apparent cause.^1^, ^3^, ^4^, ^5^ Its etiology remains unclear.^7^ It must be differentiated from other anterior mediastinal masses (including thymic lymphoma, thymoma, and germ cell tumors) and from other causes of thymus enlargement (thymus lymphofollicular hyperplasia typical of myasthenia gravis^3^, ^8^, ^9^ or thymus enlargement after treatment for malignant tumors, stress, and steroid therapy).^1^, ^5^, ^6^, ^7^, ^10^ Clinical and instrumental diagnosis is difficult. Chemical shift magnetic resonance imaging is reported to be helpful in differentiating thymic lymphoid hyperplasia from thymic neoplasm, but it is not always enough.^11^ Therefore, separating these entities requires fine‐needle aspiration or biopsy.^1^, ^2^, ^3^, ^4^, ^5^, ^10^

Massive true thymic hyperplasia (MTH) is a variant of TTH. As there are no generally accepted criteria for defining “massive,” in literature, the following guidelines are suggested: (a) the thymus should be greater than the heart shadow on posterior‐anterior chest radiograph, (b) it should weigh several times the expected weight for the age of the patient, and (c) it should represent more than 2% of the body mass.^1^, ^2^, ^5^ About 50 cases of MTH have been recorded.^5^, ^10^, ^12^ The majority of cases occur between 1 and 15 years, rarely later.^5^

Clinically, MTH could present with effects of mediastinal compression, for instance, respiratory distress, dysphagia or airway obstruction, acute or recurrent pulmonary infections, or less commonly as an incidental finding.^4^

Being a rare condition, there are no guidelines on its management.A review of the literature revealed that most patients (80%) were managed surgically with complete excision of the mass, with no postoperative complications.^4^ Some authors reported that a course of steroid therapy could decrease the size of an enlarged thymus gland, because of its immunosuppressive effect on T and B lymphocytes that mature in the thymus gland particularly in infancy.^4^, ^13^, ^16^ But in MTH there is no evidence of efficacy; some of these masses do not respond or continue to growth after steroid therapy ends, sometimes to sizes greater than pretreatment.^2^, ^4^, ^13^, ^14^, ^15^, ^17^, ^18^, ^19^, ^20^, ^21^ Both normal thymic tissue and lymphomas may show rebound growth after the suspension of steroids.^4^, ^13^, ^19^, ^20^, ^21^ Furthermore, there are no clear indications regarding duration and dosage of the corticosteroid. A small number of patients were reported to be treated conservatively, mostly for incidental findings, or in children with few symptoms and with no acute complications.^4^, ^10^ In fact, the rate of atrophy of an MTH is often very slow and it could potentially lead to complications such as acute airway obstruction.^4^ This is true especially in infants, who are furthest from the time of the physiological involution of the thymus and when the thymus has the largest relative size.^1^

In our case, the decision as to the best management was not easy to take, because of the few cases described and the absence of clear guidelines on therapy. Following confirmation of TTH by biopsy, a period with steroid therapy was tried. It proved ineffective, with subsequent enlargement of the mass. Subsequently, we performed total surgical excision of the mass easily and with no complications.

With the aim of analyzing clinical presentation and management of giant thymic hyperplasia in young infants, we reviewed the cases of MTH reported in the literature between 1976 and March 2018 in children aged <2 years of life, the age when the thymus is relatively largest.^1^, ^6^

A total of 14 cases met the inclusion criteria, and they are all reported in Table 1.^1^, ^2^, ^3^, ^4^, ^5^, ^6^, ^7^, ^12^, ^14^, ^17^, ^18^, ^22^, ^23^

**Table 1:** A total of 14 cases of MTH in children <2 y old reported in literature, including our index case

No.	Ref.	Sex	Age	Weight (kg)	Presenting symptoms	Treatment	Thymus size	Surgical complications	Outcome (follow‐up reported)
1	O Shea et al (1978)^17^	M	1 y	‐	Respiratory distress	1. Steroid (regrowth after therapy suspension) 2. Surgical thymectomy	420 g	No	Asymptomatic (5 mo)
2	Lamesch (1982)^13^	F	7 mo	5.7	Respiratory distress	1. Steroid and ventilation (ineffective) 2. Surgical thimectomy	230 g 18 × 11 × 8.5 cm	No	Asymptomatic (7 y)
3	Parker et al (1984)^6^	M	15 mo	‐	Respiratory tract infection	Surgical thimectomy	200 g	No	Asymptomatic (not listed the time of follow‐up)
4	Linegar et al (1993)^4^	F	2 mo	3.6	Chest infection, respiratory distress, acute airway obstruction, splenomegaly	Surgical thimectomy	220 g	No	Asymptomatic (3 mo)
5	Lee et al (1996)^1^	M	11 mo	8	Fever and upper respiratory symptoms	Surgical thimectomy	500 g	No	Asymptomatic (not listed the time of follow‐up)
6	Szarf et al (2010)^23^	M	2 y	‐	Respiratory infections and persistent tachyonoea	Surgical thimectomy	830 g	No	Asymptomatic (not listed the time of follow‐up)
7	Tan et al (2010)^2^	F	9 mo	8	Fever and upper respiratory symptoms	1. Steroid (2 mg/kg/d for 2 wk, unsuccessful) 2. Surgical thymectomy	200 g 17.5 × 11 × 5 cm	No	Asymptomatic at the discharge from hospital (fu not listed)
8	Katz et al, (1977)^12^	M	7 mo	‐	Hepatomegaly	surgical thimectomy	224 g 9 × 8 × 6 cm	No	Asymptomatic (4 y)
9	Lee et al (1979)^18^	F	22 mo	11.8	Asymptomatic (incidental finding)	1. Steroid (prednisone 1.5 mg/kg/d for 5 d, unsuccessful) 2. Surgical thymectomy	550 g 19 × 12 × 4.5 cm	No	Asymptomatic (6 mo)
10	Weis et al (2017)^13^	M	1 mo	‐	Respiratory insufficiency, cardiocirculatory instability	1. Steroid (prednisolone at high doses for 12 d, ineffective) 2. Surgical thymectomy	200 g 8.5 × 3.8 × 7.5 cm	No	Recurrence[Link] → 2nd resection → asymptomatic (1 y)
11	Regal et al (2007)^5^	M	5 mo	‐	Respiratory distress	Surgical thymectomy	380 g	No	Asymptomatic (2 y)
12	Woywodt et al (1999)^7^	M	11 mo	‐	Pneumonia	Surgical thymectomy	550 g 17 × 5 × 3 cm	No	Asymptomatic (6 y)
13	Sayed et al^22^	M	3 mo	3.5	Respiratory distress, failure to thrive	Surgical thymectomy	219.7 g 12 × 14 × 5 cm	No	Diagnosis of BWS, hepatic hemangioma
14	Our case index	M	15 mo	10.5	Respiratory infection	1. Steroid (prednisone 2 mg/kg/d for 1 mo, ineffective) 2. Surgical thymectomy	492 g 15 × 11.5 × 5 cm	No	Asymptomatic (10 mo)

The first column of the table refers to the cases of MTH included in our review, with the reference of the literature. The 2nd, 3rd, and 4th columns refer, respectively, to gender, age, and weight (if listed) of patients. The 5th, 6th, 7th, and 8th columns report symptoms at onset, management, mass size, and surgical complications. The last column refers to the outcome.

(M, male; F, female. y, years; mo, months; d, day. BWS, Beckwith‐Wiedemann syndrome)

Recurrence of TTH at short distance from the first surgery. A second surgical resection was performed, and then the child has been asymptomatic in the follow‐up at 1 y.

Ten out of fourteen (71.4%) were male and four out of fourteen (28.6%) female. The median age at onset of symptoms was 10.3 months old. Twelve out of fourteen (85.7%) patients presented with respiratory symptoms. MTH was discovered incidentally in only one patient. Six out of fourteen (42.9%) patients were initially given steroid treatment, with no benefits: in one case, the thymus regrew after therapy was suspended; in the others five cases, it was ineffective also due to significant preoperative shrinkage.

Finally, all patients, including our case, were managed surgically. In children aged <2 years of age with MTH, no conservative treatment has been reported. No peri‐ or post‐operative complications have been observed. In our case, the giant mass was well‐distinguished from the surrounding tissues and was not infiltrating; it was easily removed.

Thirteen of the fourteen (92.9%) of patients were asymptomatic during follow‐up. Only in one case, a recurrence was observed during follow‐up and it was treated successfully with a second surgical resection.

## CONFLICT OF INTEREST

Neither this paper nor any part of its essential substance has been or will be published or submitted to another scientific journal or is being considered for publication elsewhere. This submission represents original work. All the authors contributed to the writing and/or revision of the manuscript, and we have all read and approved the submission of this manuscript. We declare no conflict of interest in relation to this paper. Parents give their consent for publication of textual material (case history) and radiographic images.

## AUTHOR CONTRIBUTION

ET, MC, and LP: revised the literature and drafted the manuscript. DD: contributed to the enrollment of the patient, diagnosis, and management. DD, GP, and AP: revised the article and approved the manuscript in the final form.
